# Effect of Fabrication Technique on the Microgap of CAD/CAM Cobalt–Chrome and Zirconia Abutments on a Conical Connection Implant: An In Vitro Study

**DOI:** 10.3390/ma14092348

**Published:** 2021-04-30

**Authors:** Pedro Molinero-Mourelle, Rocio Cascos-Sanchez, Burak Yilmaz, Walter Yu Hang Lam, Edmond Ho Nang Pow, Jaime Del Río Highsmith, Miguel Gómez-Polo

**Affiliations:** 1 Department of Conservative Dentistry and Orofacial Prosthodontics, Faculty of Dentistry, Complutense University of Madrid, 28040 Madrid, Spain; rcascos@ucm.es (R.C.-S.); jrh@odon.ucm.es (J.D.R.H.); mgomezpo@ucm.es (M.G.-P.); 2 Department of Reconstructive Dentistry and Gerodontology, School of Dental Medicine, University of Bern, 3007 Bern, Switzerland; burak.yilmaz@zmk.unibe.ch; 3 Department of Restorative, Preventive and Pediatric Dentistry, School of Dental Medicine, University of Bern, 3007 Bern, Switzerland; 4 Prosthodontics, Restorative Dental Sciences, Faculty of Dentistry, The University of Hong Kong, Sai Ying Pun, Hong Kong, China; retlaw@hku.hk (W.Y.H.L.); ehnpow@hku.hk (E.H.N.P.)

**Keywords:** dental implant, dental implant–abutment design, implant–abutment interface, dental implant abutment connection, microgap

## Abstract

The aim of this in vitro study was to investigate the microgaps at the implant–abutment interface when zirconia (Zr) and CAD/CAM or cast Co–Cr abutments were used. Methods: Sixty-four conical connection implants and their abutments were divided into four groups (Co–Cr (milled, laser-sintered and castable) and Zirconia (milled)). After chewing simulation (300,000 cycles, under 200 N loads at 2 Hz at a 30° angle) and thermocycling (10,000 cycles, 5 to 50 °C, dwelling time 55 s), the implant–abutment microgap was measured 14 times at each of the four anatomical aspects on each specimen by using a scanning electron microscope (SEM). Kruskal–Wallis and pair-wise comparison were used to analyze the data (α = 0.05). Results: The SEM analysis revealed smaller microgaps with Co–Cr milled abutments (0.69–8.39 μm) followed by Zr abutments (0.12–6.57 μm), Co–Cr sintered (7.31–25.7 μm) and cast Co–Cr (1.68–85.97 μm). Statistically significant differences were found between milled and cast Co–Cr, milled and laser-sintered Co–Cr, and between Zr and cast and laser-sintered Co–Cr (*p* < 0.05). Conclusions: The material and the abutment fabrication technique affected the implant–abutment microgap magnitude. The Zr and the milled Co–Cr presented smaller microgaps. Although the CAD/CAM abutments presented the most favorable values, all tested groups had microgaps within a range of 10 to 150 μm.

## 1. Introduction

Single implant restorations for partially edentulous patients are a reliable and predictable treatment option with high medium- and long-term survival and success rates [1,2]. Nevertheless, these reconstructions can present with biological peri-implant complications and/or screw, abutment, and implant-related technical complications [2,3]. Among other technical complications, implant–abutment misfit can cause stresses at the connection area, lead to screw and abutment loosening or fracture, and implant overloading [4,5]. This situation can also result in microleakage and bacterial colonization, which can lead to a consequent peri-implant pathology [6,7,8]. Several factors can lead the implant–abutment microgap formation, which can be the consequence of poor material selection, a deficient material quality, an imprecise design or manufacturing technique, manipulation of engaging areas and the use of non-original prosthetic components [9,10,11,12].

Several alternatives have been proposed in order to improve the seal in implant–abutment complex capacity, such as conical connection implants, computer-aided design/computer-aided manufacture (CAD/CAM) processes, or the use of original components [13,14,15].

Considering the implant connection types, the external hexagonal connection has been conventionally most widely used and reported in the literature for the microgap evaluation. However, varying internal connection types such as internal hexagon, tri-channel connections or more recently conical connection allows for a more favorable seal between the prosthetic component and the implant connection, thereby avoiding unwanted micro-movements or micro-leakage [13,14]. In addition, the use of the CAD/CAM technology allows one to fabricate prosthetic components with greater precision, since those are based on digital designs from the same implant connection metric. Therefore, the error range is minimal and the microgap existence is lower [11,12,15].

Single implant restorations are traditionally cast in precious alloys. However, with the increasing gold price, non-precious alloys such as cobalt–chrome (Co–Cr) are widely used instead [16,17,18]. Co–Cr is a common prosthetic material with good biomechanical properties, high elastic moduli, elastic limit and a resistance to fracture. In addition, it presents a high corrosion resistance which has been shown to be effective in the salivary medium, and the material is affordable [18]. With the advent of CAD/CAM technology, new fabrication techniques such as milling and laser sintering are now available, which can largely reduce the time and cost with better quality control when compared with the conventional workflow [18,19]. Co–Cr can be processed by using casting, laser sintering (LS) or milling to fabricate prosthetic components [19]. The conventional casting technique has been commonly used, and although it is relatively affordable, the outcomes depend, to a great extent, on the technician’s skills for the design and on the quality of the alloy used [18]. The laser sintering technique is based on CAD/CAM technology, reduces manufacturing time and costs, minimizes human errors and prevents possible defects in cast prosthetic components without material waste [20]. The milling technique has similar advantages to laser sintering in terms of fabrication performance and cost-efficacy and the finish and precision obtained has a smoother surface; however, the material waste with the milling technique is higher [21].

On the other hand, ceramic materials have also recently become popular due to the global demand for metal-free restorations [16,17,18]. Among all ceramic materials, zirconia (Zr) is the most commonly used abutment/crown material in implant-prosthodontics. In addition to its good mechanical properties, it has biocompatibility and favorable esthetics. Zr is commonly used with titanium bases (Ti-base) or made into a one-piece abutment crown [16,17,22]. According to the results of a recent systematic review, Zr abutments are a reliable alternative presenting clinical advantage, providing favorable color matching and soft tissue texture, particularly when the soft tissue is a phenotype thinner than 2 mm [23].

There has been a dramatic increase in the number of single implant crowns fabricated by using conventional and digital workflows in the last decade [24]. However, studies which have evaluated the fit of Co–Cr abutments fabricated with different techniques are lacking. Therefore, the aim of the present in vitro study was to assess the microgaps at the abutment–implant interface when cast, milled, or laser-sintered Co–Cr abutments and Zr abutments were used. The null hypothesis was that the microgaps with cast Co–Cr, laser-sintered Co–Cr, and milled zirconia abutments would not be significantly different than those with milled Co–Cr abutments (control).

## 2. Materials and Methods

### 2.1. Study Design

A total of 64 implant abutments were designed and fabricated in one-piece by using different techniques in 4 groups (*n* = 16). The sample size was determined using the nQuary software (nQuery Avisor Sample size, version 8.5.2; Statistical Solutions Ltd., Cork, Ireland). A minimum of 12 specimens were required in each group for a power of 80% based on previous studies [25] assuming a common standard deviation of 3.5, by using a two-group t-test with a two-sided significance level of 0.05.

### 2.2. Specimen Preparation

**Figure 1:**
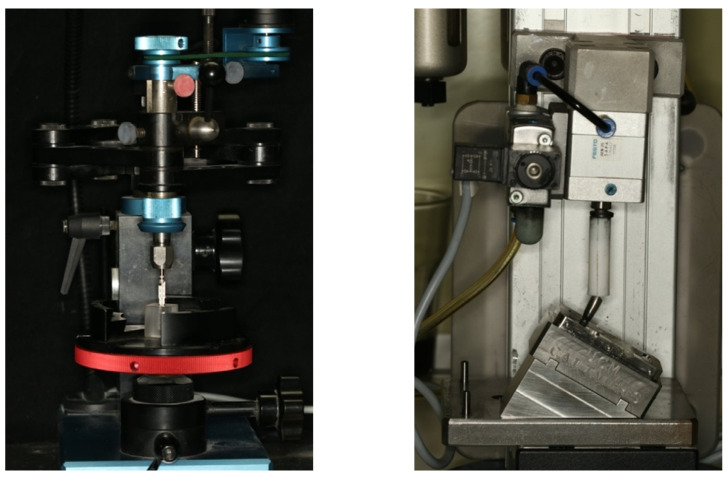
Parallelizing drill press with the implant system drill for the osteotomy preparation (**Right**); Chewing simulation machine (Instron^®^, Euroortodoncia S.A. and Complutense University of Madrid), (**Left**).

First, the osteotomies (*N* = 64) were prepared in a polymethylmethacrylate resin block (1.8 mm length and 1.5 mm width, (Mechanical Workshop, Faculty of Physics, Complutense University of Madrid)) with an elastic modulus of 3000 MPa (cancellous bone approximate module of 1.507 MPa) [26]. A parallelizing drill press was used to standardize the implant position/angle, and the implants (4.1 mm × 8.5 mm, Ocean, Avinent implant system, Santpedor, Barcelona, Spain, Ref. n° 1590 (*n* = 64)) were placed using a surgical handpiece (iChiropro) with Micro-Series CA 20: 1 L (Bien-Air Dental SA, Bienne, Switzerland) following the manufacturer’s protocol. Implants were tightened to 35 Ncm manually with the help of the implant system’s rachet, leaving the implant platforms at the resin level (Figure 1).

In each group, 16 identical abutments with one-piece crown configuration were fabricated following the design of a mandibular first premolar as the customized master abutment. An abutment analog (4.1 mm) was placed in an autopolymerizing acrylic resin base (CandiQuick, ScanDia, Hagen, Germany), and a laboratory scan body was tightened (Core 3D centers, Dental Direkt GmbH, Spenge, Germany). The scan bodies were scanned with a laboratory scanner (Biomet Zfx, Zimmer ZFX, GmbH, Dachau, Germany) and a standard triangle language (STL) file was generated. The STL file was exported to the Exocad CAD software (Exocad GmbH, Darmstadt, Germany). The master abutment was digitally designed. Two different materials were used for the fabrication of crowns; milled 3 mol% yttria stabilized tetragonal zirconia polycrystalline doped with alumina (3Y-TZP-LA) and cobalt–chromium (Co–Cr) by using three different techniques (milled, cast and laser sintering).

### 2.3. Abutment Fabrication

The material compositions and manufacturer information are displayed in Table 1. 3Y-TZP-LA (ZrCAD) abutments were fabricated by a processing center with a license from the implant manufacturer using the digital library of original components for its design following the specifications of the International Organization Standard (ISO) 13356:2015 on yttria-stabilized tetragonal zirconia [27] with a 5-axis precision milling machine (Roland DWX-52DC; Roland DG Deutschland GmbH, Willich, Germany) (G1). Co–Cr abutments were fabricated by using 3 different techniques (milled, laser-sintered, cast) following the specifications of the ISO standard 583-12:2019 for Co–Cr [28].

The Co–Cr milled group (G2) was fabricated by the same center, which fabricated the zirconia abutments in a 5-axis milling machine (DMG Sauer HSC 20 Linear (DMG MORI AKTIENGESELLSCHAFT, Bielefeld, Germany). The laser-sintered abutments (G3) were fabricated in a selective laser melting machine (SLM 125; SML Solutions Group AG, Lubeck, Germany) following the same CAD design, by using following laser sintering printing parameters: layer thickness (LT): 0.3 mm; laser spot size (LSS): 365–415 nm; laser power (LP): 400 W; type of supports (TS): pines.

For the cast group (G4), to fabricate the same premolar design used in CAD, one of the frameworks previously made was introduced in an additional silicon block in its heavy form (Platinum 85 TOUCH, Zhermack SpA, Rome, Italy), where the wax was injected into to prepare the wax patterns for the lost-wax technique. Subsequently, the wax patterns were melted to a plastic castable abutment (Dental Smart Solutions, Terrats Medical S.L. Barberá del Valles, Spain), and the Co–Cr abutments were cast by using induction heating and centrifugal casting in a protective gas atmosphere. The abutments were tightened to 35 Ncm by using implant system’s brand-new wrench (Avinent implant system, Santpedor, Barcelona, Spain).

### 2.4. Aging Processess

All specimens were subjected to cycling loading in a chewing simulation machine (Instron^®^, Euroortodoncia S.A. and Complutense University of Madrid. Zwick/Roell testXpert ll Software) for 300,000 cycles, under 200 N loads at 2 Hz at a 30° angle [29] in a room with a stable temperature between 20 and 22 °C and relative humidity always less than 80% (Figure 1). The load was applied with a 2 mm polytetrafluoroethylene (PTFE) cylinder on the occlusal surface of the abutments. After cyclic loading, thermocycling (10,000 cycles, 5 to 50 °C, dwelling time 55 s) in a custom-made thermo-cycling machine (Complutense University of Madrid, Spain) was performed in self-made artificial saliva.

After chewing simulation and thermocycling, before the microgap analysis, all specimens were visually inspected for failure by using 2.5× magnification loupes (ExamVision ApS, Samsø, Denmark), and a tactile motion test was performed with dental pliers. After inspection, all specimens were cleaned by using a polishing set for metal and ceramic (Komet Dental, Gebr. Brasseler GmbH and Co. KG, Lembo, Germany) and stored in distilled water for 24 h in an airtight glass container for further evaluation.

### 2.5. Microgap Definition and Assessment

**Figure 2:**
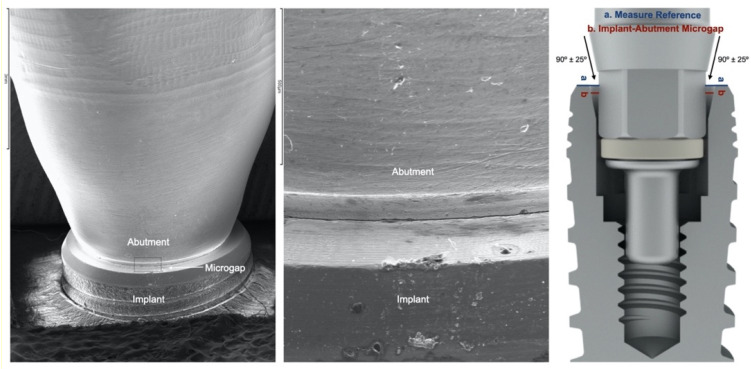
Co–CrMill abutment, SEM misfit assessment scheme, and microgap measurement concept.

The microgap was defined as angular misfit (difference between the inclined angles of the implant/abutment and the framework cylinder), or the discrepancy/lack of contact measured from the implant platform to the abutment’s corresponding contact area as had been performed in previous gap evaluation studies with internal and conical connection implants (Figure 2) [19,30,31,32]. The microgap was assessed by using a scanning electron microscope (SEM (JSM-6400, JEOL, Tokyo, Japan)) by a microscopy specialist (ICTS National Electron Microscopy Centre; University Complutense of Madrid, Madrid, Spain).

Before the measurements, the specimens were coated with 24 Kt, 19.32 g/m^3^ density gold using the Q15RS metallizer (Quorum Technologies, Sussex, UK).

**Figure 3:**
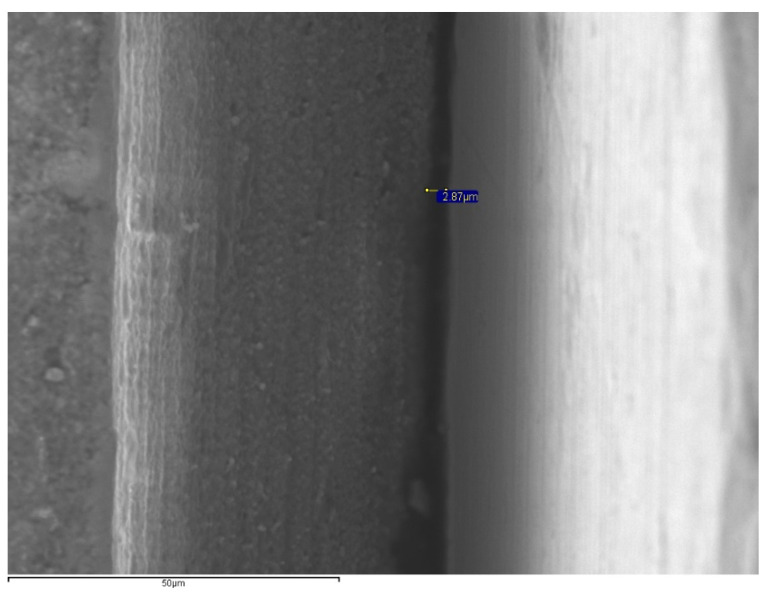
SEM images for microgap assessment (1000×) of ZrCad (2.8 μm).

**Figure 4:**
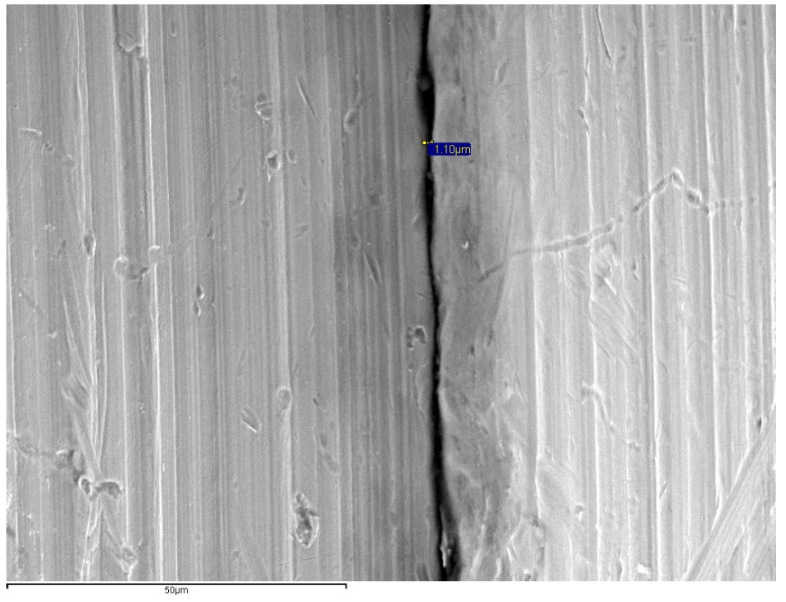
SEM images for microgap assessment (1000×) of Co–CrMill (1.10 μm).

**Figure 5:**
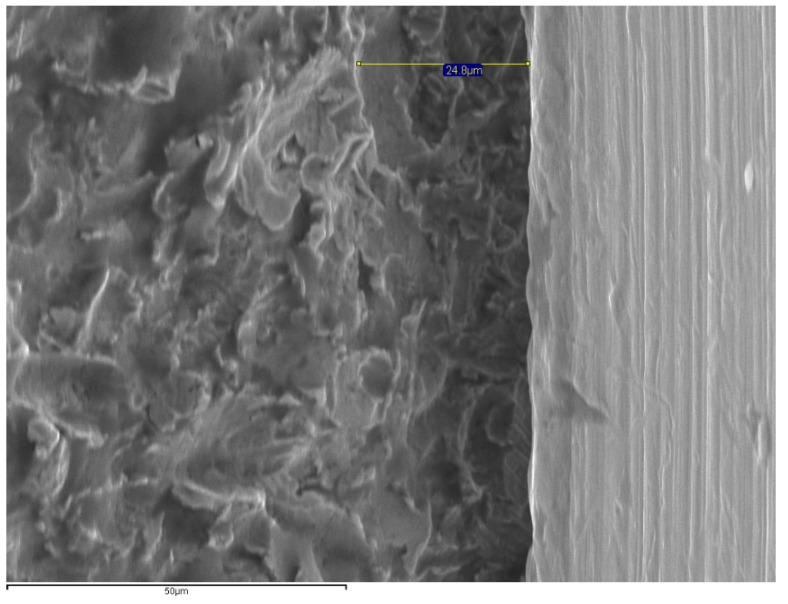
SEM images for microgap assessment (1000×) of Co–CrLS (26.9 μm).

**Figure 6:**
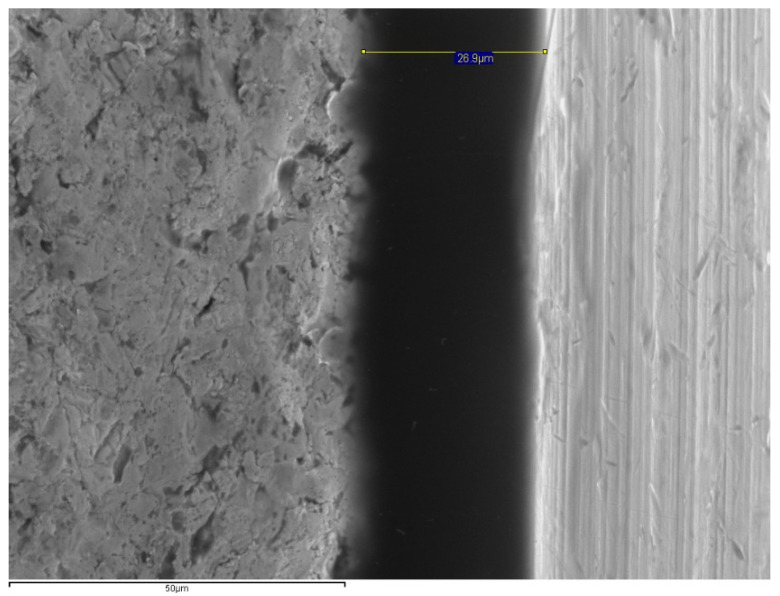
SEM images for microgap assessment (1000×) of Co–CrCL (24.8 µm).

The specimens were positioned perpendicular to the optical axis of the SEM and the microgap (90° ± 25°) (Figure 2) was assessed at 4 points of each abutment, marked with a permanent marking pen [19,30]. The distance measurement was made parallel to the axis between the implant and the abutment, at the marked points of the vestibular, mesial, distal and lingual aspects at 1000× magnifications (Figure 3, Figure 4, Figure 5 and Figure 6).

The fit was measured at 64 points (4 per specimen) in each group that were equally distributed on each image using the SEM’s INCA software (INCA microanalysis suite 4.04, Oxford Instruments, Abingdon, UK) (Figure 3, Figure 4, Figure 5 and Figure 6).

Due to the limitations with the software program, which allowed only one measurement per image, 13 more measurements were made at each point of each sample by using a the ImageJ64 software (ImageJ64, National Institutes of Health, version J64 Bethesda, MA, USA) by an independent researcher to reduce the operator bias (P.M-M.). Published recommendations were followed to enhance the reliability of the measurements by increasing the number of measurements, which required a minimum of 50 measurements for each restoration [33,34]. Fourteen measurements were made per aspect, making a total of 56 per sample, 896 per restorative group, and a total of 3584 measurements were recorded for the analysis.

### 2.6. Statistical Analysis

Data analysis was performed by using the SPSS statistical software (SPSS V25.0; IBM Corp, Armonk, NY, USA) considering the Co–Cr milled group as the control. The mean microgap and standard deviations were calculated for each aspect and in total. The Shapiro–Wilk test was used to evaluate the distribution of the data. Due to the non-normal distribution, the nonparametric Kruskal–Wallis test with the Bonferroni–Holm correction was used to analyze the microgap differences among the groups. Although the control group was Co–Cr milled, comparisons were made among all included groups. *p* values smaller than 0.05 were considered statistically significant.

## 3. Results

No complications occurred during the chewing simulation and thermocycling; therefore, all specimens were included in SEM microgap evaluation. Per aspect/point (buccal, lingual, mesial and distal) and overall means and standard deviations of microgaps in assessed groups are summarized in Table 2 and Table 3, respectively. The mean microgap value range was 0.12–6.57 μm for ZrCAD, 0.69–8.39 μm for Co–CrMill, 7.31–25.73 μm for Co–CrLS, and 1.68–85.97 μm for Co–CrCL (Figure 4). The smallest microgap was measured in the Co–CrMill group (2.26 ± 1.96), followed by the ZrCAD group (2.57 ± 1.54) both being significantly lower (*p* < 0.05) than the misfit of the Co–CrCL and Co–CrLS groups. The Co–CrCL group presented the highest mean microgap (18.40 ± 22.89), which was significantly higher than the ZrCAD and CoCrMill groups (*p* < 0.05). No significant differences were found when ZrCAD was compared with CoCrMill, and the Co–CrLS was compared with Co–CrCL (*p* > 0.05). The Kruskal–Wallis test showed significant differences between Co–CrMill and Co–CrCL, Co–CrMill and Co–CrLS, ZrCAD and Co–CrCL, and ZrCAD and Co–CrLS (*p* < 0.05) (Table 3).

## 4. Discussion

The null hypothesis was rejected since significantly different microgap values were found among groups. Although the materials and fabrication techniques for implant-supported restorations has been significantly improved in last 30 years, implant framework misfit still exists, which is considered to be an important parameter determining the long-term success of implant-supported restorations [5].

The implant–abutment microgap measurement method could influence the results. In the present study, the direct measurement of the misfit at the junction between the abutment and the implant platform, as previously described, was performed to avoid possible bias [19,30,32]. For the reliability of measurement methods, some studies recommended a minimum of 50 measurements [33,34,35,36], and some proposed up to 100 measurements per specimen [22,26]. In accordance with previous recommendations, 56 marginal evaluations were made for reliable misfit measurements in the presented study.

It has been reported that the presence of discrepancies and gaps at the junction of abutments and implants is inevitable [37,38]. However, an optimal fit is essential and should be considered when designing and fabricating these components [39]. The reported acceptable marginal microgap between the prosthetic framework and the dental implant has been changing [40] over time ranging from 30 μm [41] to 150 μm [42] since the initial studies in implant dentistry. Currently, there is a lack of consensus on the definition of acceptable misfit [5]; a recent systematic review reported that an acceptable maximum misfit could be considered as 150 μm, however, without substantial scientific evidence [30].

Some recent studies considered the gap size of 1 to 49 μm as acceptable in a preclinical and clinical scenario [19,43]. In addition to the gap size, it should be noted that the mean size of periopathogenic bacteria is around 0.2 to 1.5 μm in width and 1 to 10 μm in length [12,19,44,45]. Considering these previously reported ranges as a reference, the misfit of all groups in the present study were within an acceptable range (10–150 μm) [30]. However, some other studies reported a gap of less than 10 μm as acceptable [9,22,44,45,46]. When these studies are considered, only the abutments fabricated by the original manufacturer (ZrCAD (1.70–3.11 μm) and Co–CrMill (1.66–3.62 μm)) were within the acceptable range while the non-original groups (Co–CrLS (11.54–16.71 μm) and Co–CrCL (16.78–19.57 μm)) were not. Therefore, it can be interpreted that the fabrication technique and material do have an effect on the implant–abutment microgap. Although the influence of the fabrication technique and material on the marginal misfit has been widely assessed [5,19,32,38,47], limited studies were performed on one specific material. Most of the studies evaluated and compared standardized (stock) components with customized components made of precious alloys, titanium, and zirconia. In addition, there is a lack of studies which have evaluated CAD/CAM Co–Cr abutments including milled, cast, and laser-sintered comparing with zirconia. In a recent study, Gonzalo et al. evaluated the microgaps of milled titanium and laser-sintered Co–Cr abutments over internal connection implants by using SEM; nevertheless, the study did not compare different Co–Cr fabrication techniques, and greater misfit was found in the laser-sintered Co–Cr (11.83 to 13.21 μm) than the milled titanium (0.75 to 1.27 μm) [19].

In a similar study, different abutment materials and types (zirconia and titanium) on conical connection implants were evaluated [38]. In line with the present study results, the smallest microgaps and better fit were reported between zirconia abutments and conical connection implants with misfit values from 2.7 to 4.0 μm [37]. In another study, Co–Cr abutment misfit using different fabrication techniques was investigated [47]. The largest misfit was found in the laser sintering group (11.30 μm) followed by the cast (9.09 μm) and then the milled group (0.73 μm) [47].

Another study evaluated zirconia and titanium abutments instead of Co–Cr and reported greater mean misfit values of 7.4 to 26.7 μm for the zirconia compared with the 2.0 to 6.6 μm of Ti-abutments [48]. Baldasarri et al. evaluated the microgap between conical connection implants and customized abutments (zirconia against titanium). The zirconia abutments had a wider mean microgap range (1.5 to 34.3 μm) compared with the presented study results, and although the milled metallic alloy tested was different, they reported similar misfit values (1 to 3.5 μm) [32].

Considering these findings, the assessment of the effect of technique and material on microgaps is currently a valid research topic in implant prosthodontics. However, additional comparative studies evaluating the misfit among different methods using original and third-party abutments are needed. Concerning implant–abutment microgaps and misfit assessment, several methods have been proposed in order to investigate the implant–abutment connection [19,49]. Although there is no standardized method, techniques such as radiographic evaluation in two and three dimensions, the cross-section of the implant and prosthetic reconstruction, and the use of optical and scanning electron microscopes have been proposed [19,50]. The use of SEM has been widely documented as a reproducible method [19] for microgap or misfit evaluation of prosthetic components. However, SEM analysis has limitations as it can only provide two dimensional images. Other imaging techniques such as digital tomography or microtomography have been proposed to overcome this limitation. However, these techniques are expensive and require specific equipment and more time for the image processing and evaluations [51]. The use of SEM for the marginal vertical microgap assessment enables the investigation of the implant–abutment connection in a non-invasive manner. In addition, SEM has been commonly in implant-prosthodontics research [19,31,32]. Nevertheless, further studies starting from the implant–abutment microgap and based on the microleakage assessment could provide valuable information in order to quantify the clinical importance of the existence of microgaps.

However, the implant–abutment microgap in conical connection implants needs to be evaluated at the axis or plane of the contact surface between the implant and the abutment. In this respect, the proper angle for the microgap evaluation should be 90° to the implant axis parallel to the cone surface. Due the prosthetic configuration of the tested setup, the SEM had to be angled ±25° in order to obtain a proper axis for the microgap assessment. Therefore, the obtained results need to be carefully evaluated considering this ±25° angulation adjustment for the microgap to be observed.

The implant–abutment microgap measurement method could influence the results, and accordingly, the present study was based on the direct measurement of the misfit at the junction between the abutment and the implant platform as previously described to avoid possible bias [19,30,32]. For the reliability of measurement methods, some studies recommended a minimum of 50 measurements [33,34,52], and some proposed up to 100 measurements per specimen [22,26]. In accordance with previous recommendations, 56 marginal evaluations were made for reliable misfit measurements in the presented study.

Although the clinical indication of one-piece Zr customized abutments is being questioned due to its mechanical or technical complication risks, such as the chipping of the veneering ceramic and abutment fracture, these abutments are widely used, especially in the esthetic zone, and a recent systematic review suggests that their use may provide better soft tissue stability and improved color match compared to the use of titanium or gold alloys for abutments in the anterior region [23]. Therefore, the microgap assessments were also performed on zirconia abutments in the presented study. The inclusion of the zirconia abutment group enabled the comparison of the one-piece milled Co–Cr group to another one-piece milled abutment group in a different material.

Regarding the aging methodology, both the use of a dry test for the artificial aging and a test under physiological saline solution is accepted [29]. In the present study, a dry test, which is accepted among the ISO standards, was performed due to the characteristics of the chewing simulation machine used regarding the load cycles used in the present study. Moreover, a thermocycling test with artificial saliva separate to simulate intraoral aging was performed. The chewing methodology was based on previous studies, which reported that a loading cycle of 240,000–250,000 in a chewing simulator machine corresponds to approximately one-year of clinical chewing function [53,54]. The selection of the number of cycles was also based on the fact that the chewing simulator allowed 100,000 cycles each time it was programmed. The applied number of cycles in the present study were within the current standards in the literature, which varied between a minimum of 1000 to a maximum of 1,200,000 cycles with 20 to 300 N loads for crowns [55].

The present research was performed as an in vitro study; therefore, it cannot provide complete clinical simulation. However, this study can serve as a preclinical investigation as the first step for a future comparative randomized clinical trial for the performance of tested groups. Considering the limitations of aging methodology, microgap SEM analysis before and after that test could provide additional information at the time of beginning the study; however, the measurement after loading is widely documented in the literature and extrapolating the methodology to a clinical setting, early complications in implant restorations could begin to appear after the one-year of function [56].

In the present study, only conical connection implants were used, and the misfit should also be evaluated in implants with varying connections. Related to the materials, adding additional groups such as titanium pre-formed, or precious alloys abutments could provide additional scenarios to make in-depth comparisons. The method used to evaluate implant–abutment microgaps in the presented study is consistent with those reported; however, a further bacteriological assessment can provide additional data for the evaluation of the effect of misfit. The microgaps can also be measured by using different methods in the future. Therefore, further research studies are needed to provide clinical evidence that helps the clinician to make a decision for the optimal abutment material and fabrication technique.

## 5. Conclusions

Within the limitations of the present study, the following conclusions can be drawn: the material selection and the fabrication technique had a significant effect on the magnitude of implant–abutment microgaps. The zirconia abutments and the milled Co–Cr abutments had a favorable fit with the conical connection implant compared with laser-sintered or cast Co–Cr abutments. Although metal alloy abutments fabricated with the conventional cast technique are still considered as the standard for fixed single implant reconstructions, the findings showed favorable results for the CAD/CAM groups, and the reported microgaps for Zr abutments were promising. All tested groups presented microgaps within the clinically accepted range of 10 to 150 μm.

## Figures and Tables

**Table 1:** Studied material groups.

Group	Material Composition	Material Manufacturer	Prosthetic Manufacturer
ZrCAD (G1) ^1^	3 mol% yttria stabilized tetragonal zirconia polycrystal (3Y-TZP-A) ^2^	Dental Direkt GmbH. Spenge, Germany	CORE 3D PROTECH, S.L.U. Santpedor. Spain
Co–CrMill (G2) ^3^	63% Co, 29% Cr, 6% Mo, <1% Nb, Si, Mn, Fe	Dental Direkt GmbH. Spenge, Germany	CORE 3D PROTECH, S.L.U. Santpedor. Spain
Co–CrLS (G3) ^4^	59% Co, 25% Cr, 9.5% W, 3.5% M, 1% Si	S and S Scheftner GmbH-dental alloys. Mainz, Germany	Prótesis S.A. Madrid. Spain
Co–CrCL (G4) ^5^	61% Co, 24% Cr, 8% W, 2.5% Mo, 1% Nb, 1% Mn, 1% Si, 1% Fe	Dentalforschung Schleicher GmbH. Riedenburg Germany	Riosa Laboratory Pozuelo de Alarcón. Spain.

^1^ ZirCAD: CAD/CAM-fabricated zirconia frameworks, ^2^ 3Y-TZP-A: yttria stabilized tetragonal zirconia polycrystalline doped with alumina, ^3^ Co–CrMill: cobalt–chromium framework milled, ^4^ Co–CrLS: cobalt–chromium framework laser-sintered-fabricated, ^5^ Co–CrCL: cobalt–chromium frameworks with castable abutments.

**Table 2:** Means and standard deviations for microgap (µm) per buccal, lingual, mesial and distal aspect/point and overall means and standard deviations for microgap (µm) of the four assessed groups.

Fabrication Technique and Material				
Data	ZirCAD	Co–CrMill	Co–CrLS	Co–CrCL
**Microgap Per Area**				
**N**	**16**	**16**	**16**	**16**
Buccal	2.45 ± 1.91	3.62 ± 4.83	11.54 ± 10.08	19.57 ± 27.25
Palatal	1.70 ± 1.41	2.02 ± 1.21	12.97 ± 15.44	18.79 ± 30.64
Mesial	3.02 ± 5.37	1.73 ± 1.60	16.71 ± 8.68	18.44 ± 25.93
Distal	3.11 ± 2.65	1.66 ± 2.10	13.17 ± 13.13	16.78 ± 25.02
**Mean Microgap**				
Mean	2.57 ± 1.54	2.26 ± 1.96	13.60 ± 5.83	18.40 ± 22.89
Median	2.32	1.35	11.13	8.18
Minimum	0.12	0.69	7.31	1.68
Maximum	6.57	8.39	25.73	85.97

ZirCAD: CAD/CAM-fabricated zirconia frameworks, Co–CrMill: cobalt–chromium framework milled, Co–CrLS: cobalt–chromium framework laser-sintered-fabricated, Co–CrCL: cobalt–chromium frameworks with castable abutments.

**Table 3:** Descriptive microgap comparison of the materials and manufacturing techniques.

Manufacturing Technique and Material	Area	Statistical Quantifying	Standard Error	Statistical Quantifying Deviation	Sig ^1^	Sig ^2^
ZrCAD vs. Co–CrMill	B	−0.656	6.582	−0.100	0.921	1.000
	M	−0.313	6.579	−0.048	0.962	1.000
	D	−10.938	6.581	−1.662	0.097	0.579
	L	−4.125	6.583	−0.627	0.531	1.000
	T	−5.875	6.583	−0.892	0.372	1.000
ZrCAD vs. Co–CrCL	B	−18.063	6.582	−2.744	0.006	0.036
	M	−21.375	6.579	−3.249	0.001	0.007
	D	−8.125	6.581	−1.235	0.217	1.000
	L	−22.313	6.583	−3.390	0.001	0.004
	T	−21.188	6.583	−3.219	0.001	0.008
ZrCAD vs. Co–CrLS	B	−24.656	6.582	−3.746	0.000	0.001
	M	−27.188	6.579	−4.133	0.000	0.000
	D	−18.813	6.581	−2.858	0.004	0.026
	L	−32.563	6.583	−4.947	0.000	0.000
	T	−27.938	6.583	−4.244	0.000	0.000
Co–CrMill vs. Co–CrCL	B	−17.406	6.582	−2.645	0.008	0.049
	M	−21.688	6.579	−3.297	0.001	0.006
	D	−19.063	6.581	−2.896	0.004	0.023
	L	−18.188	6.583	−2.763	0.006	0.034
	T	−27.0.63	6.583	−4.111	0.000	0.000
Co–CrMill vs. Co–CrLS	B	−24.000	6.582	−3.646	0.000	0.002
	M	−27.500	6.579	−4.180	0.000	0.000
	D	−29.750	6.581	−4.520	0.000	0.000
	L	−28.438	6.583	−4.320	0.000	0.000
	T	−33.813	6.583	−5.136	0.000	0.000
Co–CrCL vs. Co–CrLS	B	6.594	6.582	1.002	0.316	1.000
	M	5.813	6.579	0.884	0.377	1.000
	D	10.688	6.581	1.624	0.104	0.626
	L	10.250	6.583	1.557	0.119	0.717
	T	6.750	6.583	1.025	0.305	1.000

ZirCAD: CAD/CAM-fabricated zirconia frameworks, Co–CrMill: cobalt–chromium framework milled, Co–CrLS: cobalt–chromium framework laser-sintered-fabricated, Co–CrCL: cobalt–chromium frameworks with castable abutments. B: buccal; M: mesial; D: distal; L: lingual; T: total. Sig ^1^: significance level at 0.05 according to Mann–Whitney U tests; Sig ^2^: Bonferroni–Holm correction for multiple comparisons.

## Data Availability

The data presented in this study are available on request from the corresponding author.
